# Rhizosphere Soil Microbial Properties on *Tetraena mongolica* in the Arid and Semi-Arid Regions, China

**DOI:** 10.3390/ijerph17145142

**Published:** 2020-07-16

**Authors:** Mengying Ruan, Yuxiu Zhang, Tuanyao Chai

**Affiliations:** 1 School of Chemical and Environmental Engineering, China University of Mining & Technology-Beijing, Beijing 100083, China; rmy@student.cumtb.edu.cn; 2 College of Life Science, University of Chinese Academy of Sciences, Beijing 100049, China; tychai@ucas.ac.cn

**Keywords:** *Tetraena mongolica*, open pit mining, soil nutrients, enzyme activities, soil microbial communities

## Abstract

*Tetraena mongolica* is a rare and endangered species unique to China. The total number and density of *Tetraena mongolica* shrubs in desertification areas have experienced a sharp decrease with increases in coal mining activities. However, available information on the *T. mongolica* rhizosphere soil quality and microbial properties is scarce. Here, we investigated the effect of coal mining on the soil bacterial community and its response to the soil environment in the *T. mongolica* region. The results showed that the closer to the coal mining area, the lower the vegetation coverage and species diversity. The electrical conductivity (EC) in the contaminated area increased, while the total nitrogen (TN), available phosphorus (AP), available potassium (AK), and soil organic carbon (SOC) decreased. The activity of NAG, sucrose, β-glucosidase, and alkaline phosphatase further decreased. In addition, the mining area could alter the soil’s bacterial abundance and diversity. The organic pollutant degradation bacteria such as *Sphingomonas*, *Gemmatimonas*, *Nocardioides,* and *Gaiella* were enriched in the soil, and the carbon-nitrogen cycle was changed. Canonical correspondence analysis (CCA) and Pearson’s correlation coefficients showed that the change in the bacterial community structure was mainly caused by environmental factors such as water content (SWC) and EC. Taken together, these results suggested that open pit mining led to the salinization of the soil, reduction the soil nutrient content and enzyme activity, shifting the rhizosphere soil microbial community structure, and altering the carbon-nitrogen cycle, and the soil quality declined and the growth of *T. mongolica* was affected in the end. Therefore, the development of green coal mining technology is of great significance to protect the growth of *T. mongolica*.

## 1. Introduction

*T. mongolica* is a rare and endangered species unique to the western Ordos Plateau in North-Central and Wuhai City, China [1]. China Red Data Book lists this rare species as the second highest priority for conservation. It is a common deciduous shrub inhabiting arid and semi-arid regions. *T. mongolica* is a xerophyte with a well-developed root system that is particularly suited to its habitat and able to cope with drought and other harsh natural conditions. It serves a role in stabilizing the sand in the region by promoting soil and water conservation and windbreaking. *T. mongolica* has been in decline in recent years. During the last 28 years, it has decreased 19.36% and covers less than 13.40 km^2^·a^−1^ [2]. This could be caused by the climate change but is attributed mainly to human activities, especially the coal mining [3].

The distribution of coal in Wuhai City is similar to the distribution of *T. mongolica*. Wuhai City mainly focuses on open pit mining. Coal mining gave rise to the destruction of the *T. mongolica* environment, where contaminants such as Cu, Pb, Mo, and As were highly enriched in the soil [4]. Furthermore, the combustion of coal particles and coal produces airborne compounds that may contain heavy metals and Polycyclic Aromatic Hydrocarbons (PAHs), which settle or wash out of the atmosphere and contaminate the soil. The normal function of the soil in mining areas is changed due to the influence of these heavy metals and polycyclic aromatic hydrocarbons [5]. Coal mining activities affect the soil’s physical quality and interfere with the diversity of soil bacterial community. Coal mining activities affect the physical quality of the soil: high bulk density and low water holding capacity, porosity, moisture content, pH, and soil nutrients (total nitrogen and available phosphorus) [6]. Coal mining activities interfered with the soil bacterial community structure, which led to a significant reduction in soil enzyme activity, with the dominant bacteria comprising Proteobacteria, Actinomycetes, and Acidobacteria [7].

Plant growth depends on nutrient cycling in the soil releasing nutrients throughout [8]. Soil nutrient determines the growth of vegetation and plays an important role in the development of ecosystem, especially the vegetation community. The diversity of soil bacterial community structure is closely related to soil function [9]. Soil bacterial community structure is the foundation of soil function. Bacteria are responsible for nutrient conversion cycle, organic matter decomposition, humus formation, and system stabilization in soil ecological functions [10]. Soil bacteria participate in soil nutrient cycling and energy flow. Rhizosphere bacterial community composition depended on plant diversity. The root activity significantly affected the biological properties of soil [11]. Until recently, research on *T. mongolica* has focused on the biological characteristics, insect-related and pollination characteristics, landscape fragmentation process, and cell and genetic structure [12,13]. However, available information on the *T. mongolica* rhizosphere soil microbial properties is scarce, especially the coal mining. In this study, the soil nutrients and bacterial community structure of soil around *T. mongolica* were measured to evaluate the change of soil quality. The objectives of the study were (1) to understand the changes of plant community structure in the *T. mongolica* regions, (2) to evaluate the variation of soil quality in *T. mongolica* soil under open pit mining conditions, (3) to investigate the effect of soil bacterial diversity near *T. mongolica* and its response to open pit mining.

## 2. Materials and Methods

### 2.1. Study Area

The study area is located in Wuhai City (39°02′30″–39°54′55″ N, 106°36′25″–107°08′05″ E), a new industrial city in the west of the Inner Mongolian autonomous region of Northwest China. There is a continental climate in the study area. The average temperature is 9.3 °C, the average annual rainfall is 162 mm, and the average sunshine exposure is 3138.6 h [14]. The vegetation coverage in the study area is 25%, and the main vegetation type is desert grassland, including such species as *T. mongolica*, *Ammopiptanthus mongolicus,* and *Zygophyllum xanthoxylum*.

**Figure 1:**
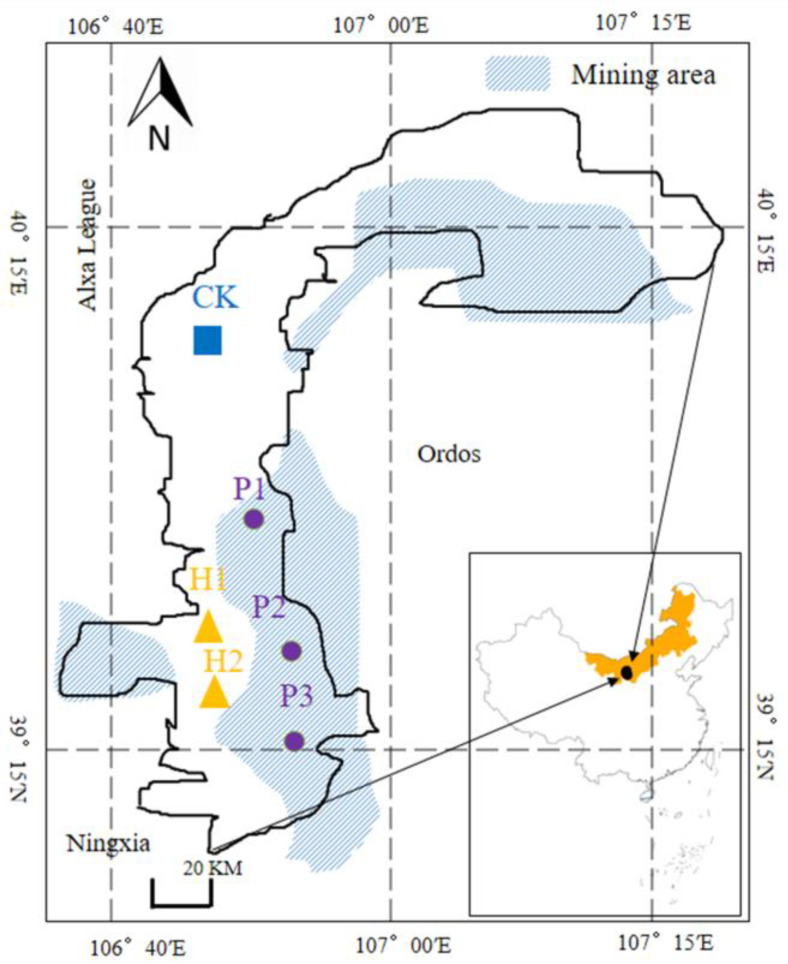
*Tetraena mongolica* distribution area and sample location.

In August 2018, based on the distribution of coal resources and *T. mongolica*, soil was collected from *T. mongolica* area disturbed by coal mining (P1, P2, P3), soil was less polluted from the Nature Reserve of *T. mongolica* (H1, H2), an adjacent unexploited area (CK). The distribution and characteristics of the samples are described in Figure 1. The soil closely bound to the roots collected within the space used by the roots is considered to be the rhizosphere [15]. In each plot, three quadrants (5 × 5 m per quadrant) were established as three soil cores. Using community ecology techniques, we investigated plant assemblages in the *T. mongolica* regions. In each quadrant, five samples were randomly collected and mixed to form a composite soil sample at soil depth (0–15 cm, 15–30 cm). Each soil sample was divided into two parts. Each part was naturally air-dried and filtered through a 2 mm mesh to test the soil properties. This portion was stored at 80 °C for subsequent high-throughput Illumina sequencing analysis.

### 2.2. Analysis of the Properties of Soil Samples

The dried soil was passed through a 1 mm sieve to determine the particle fractions. A conductivity meter was used to measure the soil electrical conductivity (EC) of the air-dried samples. Soil water content (SWC) was determined by drying soil samples at 105 °C for 24 h. The total nitrogen (TN) was determined by the Kjeldahl method [16]. The level of available phosphorus (AP) in the soil was determined by the molybdenum-blue method. The available potassium (AK) was extracted, and the level was determined using the methods described by Bao [17]. The soil organic carbon (SOC) content was determined using dichromate oxidation [18]. Mp-Nitrophenyl-N-acetyl-β-D-glucosaminide, disodium phenyl phosphate colorimetry, and indophenol-blue colorimetry were used to measure N-acetyl-β-d-glucosidase (EC 3.2.1.52, NAG), alkaline phosphatase (EC 3.1.3.1), and sucrase (EC 3.2.1.26) activities, respectively [19]. The soil β-glucosidase (EC3.2.1.21) level was determined by culture and spectrophotometry. Three repeated experiments were carried out for the above indicators.

### 2.3. Soil Microbial Community Analysis

According to the manufacturer instructions, 500 mg of soil genomic DNA was extracted from each sample using the E.Z.N.A. soil DNA kit (Omega Biotek, GA 30071, USA). The extracted DNA was purified and quantified by a NanoDrop 2000 spectrophotometer (Thermo, MA 02451, USA). Each extracted 16S rRNA v3-v4 region was amplified by PCR using the following procedure: at 95 °C for 2 min, followed by 25 cycles of denaturation for 30 s at 95 °C, annealing for 30 s at 55 °C, and extension for 45 s at 72 °C, and then 10 min at 72 °C. The primers 341F (5’-barcode-CCTACGGGNGGCWGCAG-3’) and 805R (5’-GACTACHVGGGTATCTAATCC-3’) were used [20]. The bar code for each primer corresponds to the unique eight-base sequence for each sample. The PCR mixture contained 5 μL of 10 x PCR buffer solution, 0.5 μL of 10 mm nucleotides, 10 ng of template DNA, 0.5 μL of each primer (50 mm), and 50 μL of ddH_2_O in total. The PCR products were determined by 2% agarose gel electrophoresis. The amplicon extracted from 2% agarose gel was purified using a PCR purification kit (Beckman, Indiana 46268, USA) and quantified using a Qubit^®^ 2.0 fluorimeter (Invitrogen, CA 92008, USA) according to the standard protocol. Paired sequencing was performed on the Illumina MiSeq platform (Shanghai Sangon Biotechnology Co., LTD., Shanghai, China) in accordance with the standard protocol (2300). The operational taxonomic units (OTUs) table was input into PICRUSt for metagenome prediction using the KEGG (Kyoto Encyclopedia of Genes and Genomes) database.

### 2.4. Data Analysis

Simpson Index, Shannon–Wiener Index, and Pielou’s Evenness Index can measure the species diversity of the plant community. Community richness index (Chao1 and ACE estimator) and community diversity index (Shannon and Simpson index) were calculated in Mothur using the high-throughput sequencing data. The Shannon and Simpson indices were employed to estimate soil microbial diversity. The raw data were collated with Excel 2016 and visualized with Origin 2017. SPSS 23.0 was applied for statistical analysis. The figures were produced with Origin 2017. Redundancy analysis (RDA) and canonical correspondence analysis (CCA) were used to determine the correlations between soil indicators among the samples using Canoco 5.0.

## 3. Results

### 3.1. Effect of Open Pit Mining on Plant Community Structure

Field investigations were conducted on the vegetation community structure in the area where *Tetraena mongolica* was distributed in Table 1. The vegetation coverage and species diversity indicators in polluted areas close to the mining area is low. The vegetation coverage and species diversity of *Tetraena mongolica*: Nature reserve is lower than that of the control area. Species number, coverage and species diversity index of plants at sampling points: the control area > Nature reserve > the contaminated area. The vegetation in the contaminated area is mainly *Reaumuria soongarica, Tetraena mongolica*, and *Achnatherum splendens.* The vegetation in the Nature reserve is mainly composed of *Tetraena mongolica* and *Zygophyllum xanthoxylon*. *Tetraena mongolica* and *Phragmites australis* are the main vegetation in the control area. Different locations have different vegetation types.

### 3.2. Effect of Open Pit Mining on Soil Quality

**Figure 2:**
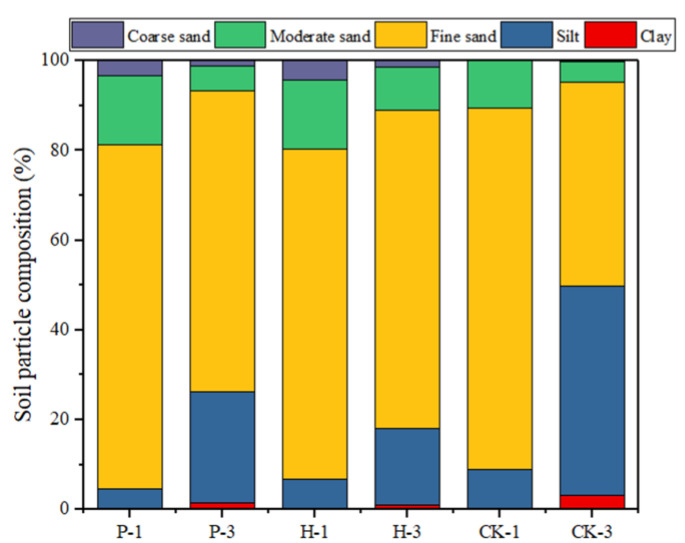
Soil particle size distribution of samples. Soil particles classifications: clay (0–5 μm); silt (5–50 μm); fine sand (50–250 μm), moderate sand (250–500 μm) and coarse sand (500–1000 μm)**.**

The soil particle size distribution is indicated in Figure 2. The contaminated area and conservation area mainly consisted of fine sand and moderate sand, where 15–30 cm is considered coarse sand. The control area mainly contained sand and silt, while sand was the major component of both the contaminated and conservation areas. The conservation area had coarse sand. There was more moderate sand and silt present in the contaminated area than in the control area. There was also less clay in the topsoil, but the amount of clay at 15–30 cm increased.

**Figure 3:**
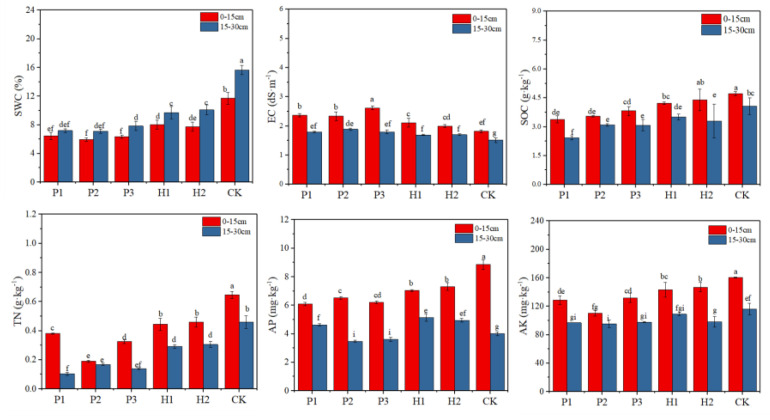
Physicochemical properties of the soil samples. Different lowercase letters indicate significant differences at 0.05 (*p* < 0.05) levels.

As shown in Figure 3, the soil SWC and EC of the samples showed different trends. The soil EC ranged from 1.80–2.62 dS·m^−1^. These soil variables gradually increased with depth, and the values in the contaminated area were statistically higher than those in the control area at the same depth. The SWC ranged from 5.95 to 15.60% in the control and contaminated areas. The SWC of the *T. mongolica* regions increased with the increase in depth. The surface water content of the contaminated area was at a low level. There were same trends in the severe group and the controls with regard to soil nutrients. For these groups, the TN, AP, AK, and SOC exhibited similar changes with depth, but the values were statistically higher in the control area than in the contaminated areas. The soil TN of all studied areas ranged from 0.11 g·kg^−1^ to 0.65 g·kg^−1^. The soil AP of all studied areas ranged from 3.48 mg·kg^−1^ to 8.91 mg·kg^−1^. The AP content decreased with the increase in depth, and the surface soil value in the control area was significantly higher than that in the contaminated area. The soil AK ranged from 95.39 mg·kg^−1^ to 160.29 mg·kg^−1^. The AK content in the topsoil was higher in the control area compared to the contaminated area. The SOC of the control areas decreased from 0–15 cm to 15–30 cm by 16.81%, while these percent decreases were 19.32% from 0–15 cm to 15–30 cm in the contaminated areas. The control areas had a lower decrease in rates over the soil depth range than the contaminated areas.

**Figure 4:**
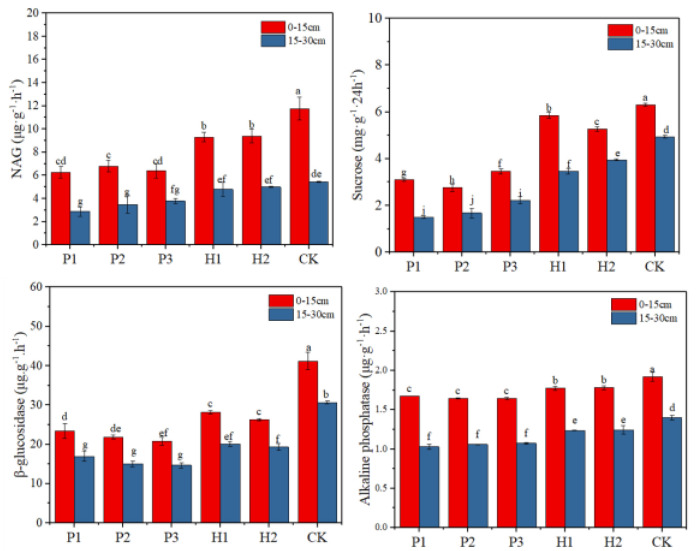
Changes in soil enzyme activities in the control and contaminated areas. Different lowercase letters indicate significant differences at 0.05 (*p* < 0.05) levels.

The activities of four enzymes (NAG, sucrose, β-glucosidase, and alkaline phosphatase) in the *T. mongolica* regions are displayed in Figure 4. Soil enzyme activity decreased with increasing depth in both the control and contaminated areas. The surface enzymes activity in the control area was significantly higher than that in other areas. Soil NAG activity in the *T. mongolica* regions ranged from 2.88 to 11.75 μg·g^−1^·h^−1^. Soil sucrose activity in the *T. mongolica* regions ranged from 1.50 to 6.31 mg·g^−1^·24 h^−1^. The activity of β-glucosidase ranged from 14.69 to 41.17 mg·g^−1^·24 h^−1^. The highest activity of alkaline phosphatase was 1.92 mg·g^−1^·24 h^−1^ in the control area at 0–15 cm depth. The activity levels in contaminated areas were significantly lower than those in the control areas. The overall activity of the four enzymes in the control area soil was significantly higher than that in the contaminated area soil. Therefore, all the results show that open pit mining reduces soil enzyme activity.

### 3.3. Effect of Open Pit Mining on Bacterial Community Structure and Composition

The coverage index actually reflects whether the sequencing results represent the real situation of the sample. The coverage of all samples was above 95%, indicating that the results of sequencing were reliable and reflected the real situation of soil bacteria. As shown in Table 2, the ACE, Shannon and Chao1 indices for six sites revealed similar trends and exhibited a gradual decrease with depth; higher values were recorded for the control area at the same depth. The Simpson diversity index was the highest among two samples collected at 15–30 cm depth, and the value of the control area was lower than that of the contaminated area. These results show that the soil structure induced by open pit mining gradually decreases the bacterial richness and diversity.

**Figure 5:**
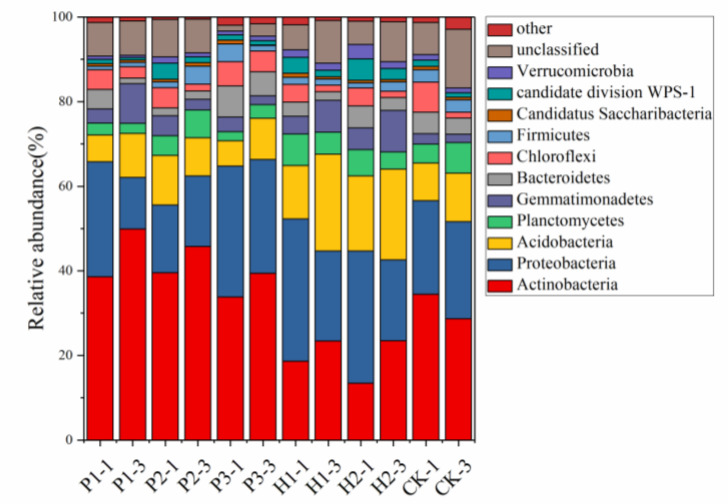
At the phylum level, soil bacterial community composition in the *T. mongolica* regions.

Bacterial 16S rRNA genes collected from 12 soil samples were sequenced by high-throughput sequencing and cluster analysis, and a total of 11 phyla were obtained (Figure 5). At the phylum level, Actinobacteria, Proteobacteria, Acidobacteria, Planctomycetes, Gemmatimonadetes, Bacteroidetes, and Chloroflexi were the 7 most abundant phyla. In addition, approximately 7% of the sequences were not assigned to any of the existing phyla, suggesting that there are unrecognized microbial resources in the region. As shown in Figure 5, the community structure of each site was the same at the phylum level, but the relative abundance of each genus was different. Actinobacteria were abundant in the contaminated areas at 15–30 cm depths, but in other areas the opposite was true. Proteobacteria were the most abundant at a soil depth of 0–15 cm. Acidobacteria were more abundant at soil depth of 0–15 cm than at soil depth of 15–30 cm. Increased levels of Planctomycetes were detected in the surface soil of the contaminated areas. Gemmatimonadetes were the most abundant at a soil depth of 0–15 cm in the contaminated area. Chloroflexi were more abundant than the deep layer when the soil depth is 0–15 cm. In the desert, Actinobacteria, Proteobacteria, Bacteroidetes, and Candidatus Saccharibacteria were the 4 most abundant phyla. Actinobacteria and Proteobacteria were more abundant at soil depth of 15–30 cm than at soil depth of 0–15 cm; they were the opposite of the levels observed for Candidatus Saccharibacteria and Bacteroidetes. At the phylum level, the contaminated area of the soil bacterial community structure was similar to the control area, and the relative abundance of bacteria belonging to each phylum exhibited obvious differences, verifying that coal mining has an apparent influence on the soil bacteria community structure.

**Figure 6:**
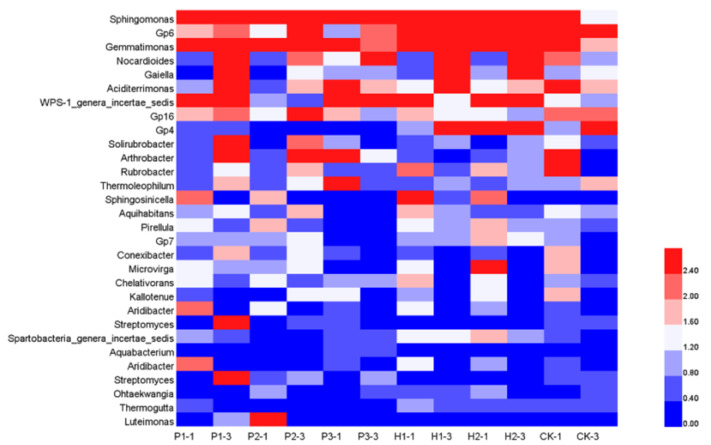
Heat map of microbial community structure in soil.

At the genus level, the thermal maps of microbial communities in different sampling areas were constructed (Figure 6). At the genus level, 628 bacterial genera were detected. The 20 most abundant genera, with a frequency >1.5% in at least one sample, were selected for heat map analysis. Based on Figure 6, the main genus types in soil samples were as follows: *Sphingomonas*, *Gp6*, *Gemmatimonas*, *WPS-1_genera_incertae_sedis*, *Nocardioides*, and *Saccharibacteria_genera_incertae_sedis*. The main genus types in the conservation area and contaminated area were *Sphingomonas*, *Gp6*, and *Gemmatimonas*. *Nocardioides*, and *Saccharibacteria_genera_incertae_sedisere* were the main genus types in the desert area. In both the control and contaminated areas, *Sphingomonas* was the primary genus, representing 2.52–8.67% the sequences. *Gemmatimonas* was the next major component of the bacterial community in all samples. *Gaiella* represented 0.45–12.37% of the sequences, among which the contaminated area harbored a significantly high percentage. *Nocardioides* represented 0.45–2.12% of the sequences obtained from the control and contaminated areas. Acidobacteria, particularly subgroups *Gp6*, *Gp4*, *Gp16,* and *Gp7*, represent approximately 10% of all bacteria found in the soil. *Saccharibacteria_genera_incertae_sedis* represented between 0.52% and 1.99% of the sequences discovered in the control and contaminated areas, respectively, among which the desert area harbored a significantly high percentage (10%). Other core genera included Pirellula, Thiobacillus, *Sphingomonas*, and Rubrobacter.

**Figure 7:**
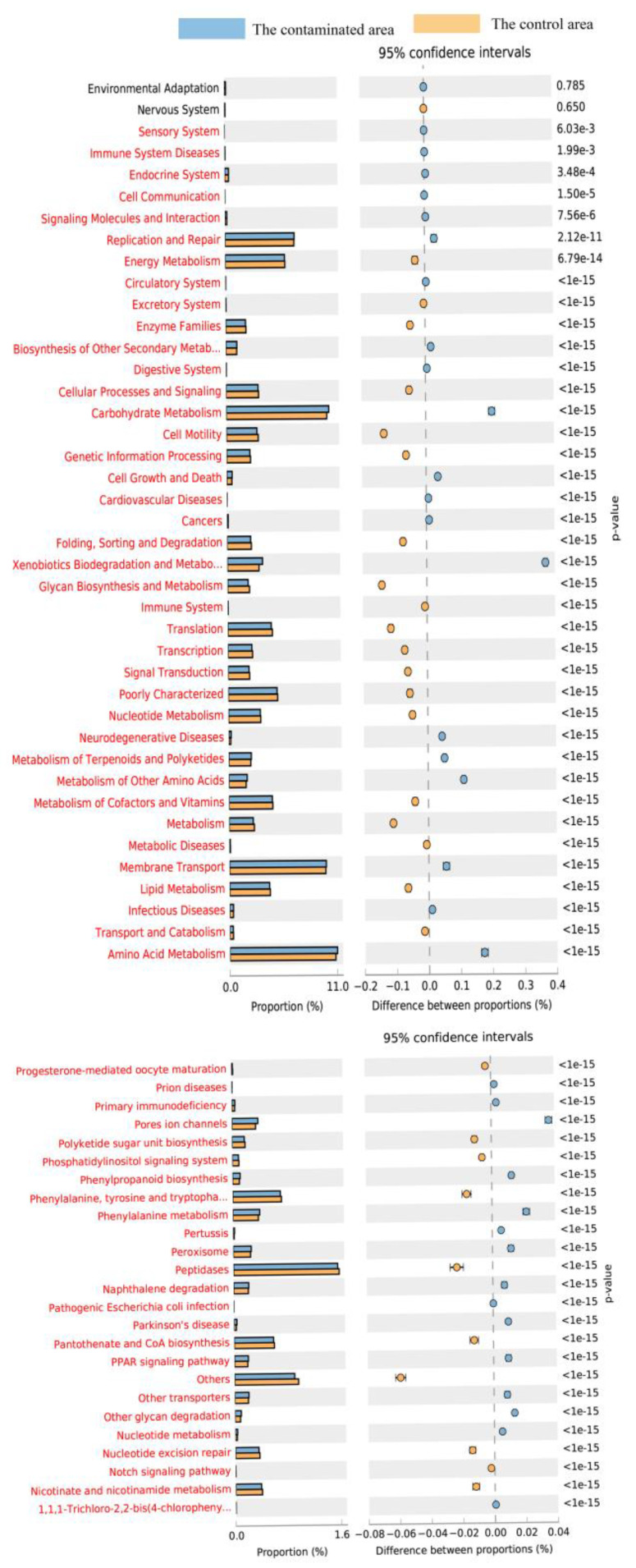
Prediction of abundance of functional gene contents of rhizosphere soil. The microbial functions were predicted at the second (**top**) and the third (**bottom**) level of the KEGG pathway.

Based on the Kyoto Encyclopedia of Genes and Genomes (KEGG) database, the abundance of functional genes is predicted. Functional genes with statistical differences at *p* < 0.05 between contaminated area and control area were shown in Figure 7. At the second level, most KEGG pathways significantly different were increased by open pit mining. The larger differences between contaminated area and control area samples were found for the terms “Carbohydrate Metabolism”, “Membrane Transport”, and “Amino Acid Metabolism”. The results indicate that coal mining causes changes in soil bacterial metabolism. At the third level, the larger differences between contaminated area and control area samples were found for the terms “Peptidases “, “Phenylalanine, tyrosine and tryptopha” and “Pantothenate and CoA biosynthesis”. Functional genes related to peptidases had enriched.

### 3.4. Response of Bacterial Community to Soil Environmental Factors

Pearson’s correlation coefficient was used to determine the relationship between soil chemical properties and soil bacterial community (Table 3). Among the main environmental factors, there is a significant relationship between every factors and soil bacterial community. The changes of eight dominant bacteria are related to soil properties. The correlation between different bacterial groups and each soil environmental factor is different. *Sphingomonas* showed a significant positive correlation with AP (*p* < 0.05); *Gp6* showed a significant positive correlation with SWC, and *Gemmatimonas* exhibited a significant positive correlation with EC and SWC (*p* < 0.05). *Nocardioides* showed a significant negative correlation with EC and SWC (*p* < 0.05), and there was a significant negative correlation with SOC and TN (*p* < 0.01). *Saccharibacteria_genera_incertae_sedis* showed a significant negative correlation with SWC and Sand (*p* < 0.05), and there was a significant negative correlation with EC, SOC, and TN (*p* < 0.01).

**Figure 8:**
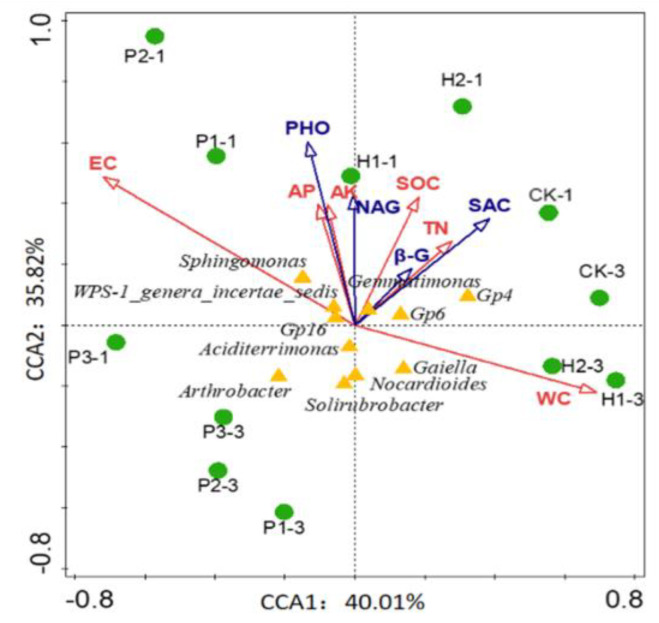
Canonical correspondence analysis (CCA) of environment variables, enzymatic activity, and bacterial diversity.

To further explore the relationships between environmental factors and bacterial community structures, a canonical correspondence analysis (CCA) tri-plot was constructed, as shown in Figure 8. CCA1 and CCA2 explained 40.01% and 35.82% of the variance, respectively. The environmental factors and soil nutrients were found to be closely related to the structure and composition of the soil microbial communities. The SWC and EC have the longest arrow lines, indicating that they are both major factors driving changes in soil nutrients and enzyme activity. Thus, the SWC and EC were the strongest determinants, respectively, with respect to the microbial community composition. AK, AP, TN, and SOC were positivity correlated with the soil bacterial community composition. Soil enzymatic activities were positivity correlated with the soil bacterial community composition.

## 4. Discussion

### 4.1. Open Pit Mining Reduced Soil Nutrient and Enzyme Activity

Open pit mining damages soil structure, decreases soil water holding capacity, and seriously affects plant growth. The closer to the coal mining area, the lower the vegetation coverage and species diversity. Although the *T. mongolica* Nature Reserve is far away from the mining area, it is also polluted, and the vegetation coverage and species diversity are reduced. In this study, we found that the particle size distribution in the contaminated area was mainly sandy soil, whereas open pit mining increases the content of sand and decreases the content of silt. The EC of soil in the contaminated area was significantly higher than that in the control area at the same depth. At the same time, the soil moisture content was low, while the soil EC value was relatively high, which reflects the desertification characteristics of poor-quality alkaline soil in this region. The SOC in the distribution area of *T. mongolica* was low, and the TN, AP, and AK were also very low. This indicates that soil fertility was very low, and the amount of nitrogen phosphorus content that could be directly absorbed by plants was very low in the contaminated areas.

*T. mongolica* regions are located in the arid and semi-arid area of Northwest China. Water is the main ecological limiting factor in the study area. The decrease in field moisture capacity may be related to the loss of physical clay in soil [21]. Severe soil erosion and wind erosion in this area are some of the most important factors causing soil desertification, with open pit mining exacerbating the problem. In arid and semi-arid areas, the rainfall rate is much slower than the evaporation rate, and the salt content accumulates in the soil and groundwater. The soil is generally alkaline, while coal mining aggravates the evaporation of water, thus causing soil salinity risk. The available nutrients in the soil are decreased in polluted areas, which may be caused by soil structure destruction due to open pit mining. Processes such as coal mining, coal transportation, coal gangue heap, discharge at will, and wind dust will intensify the accumulation of heavy metals in the surrounding soil, and this will have an impact on soil nutrients [22]. Soil enzyme activity is a sensitive and reliable index of soil biological activity and soil fertility [23,24]. For instance, NAG and phosphatase participate in soil the C, N and P cycles, which may affect soil fertility [25]. β-glucosidase can change cellulose, starch, and sugar into low molecular weight compounds [26]. Urease plays a vital role in the hydrolysis of urea into carbon dioxide and ammonia [27]. Phosphatase catalyzes organophosphate mineralization and immobilization processes, affecting the potential supply of organophosphorus. There are many factors affecting the enzyme activity, and its mechanism is complex. Soil water is one of the main factors affecting soil enzyme activity, which can regulate soil microbial activity by affecting soil osmotic potential, nutrients, energy transfer, and microbial cell metabolism, thereby affecting soil enzyme activity [28]. The soil nutrient content and enzyme activity were significantly different in among separate farmlands, and the soil urease, phosphatase and catalase activity were significantly linearly correlated with the soil organic matter and nutrient content [29]. The catalase, polyphenol oxidase, and sucrase activities in the soil were significantly correlated with the available nitrogen in the soil and that urease and polyphenol oxidase were significantly correlated with the total nitrogen content, with the sucrase activity significantly correlated with the level of organic matter present [30].

As pollution increases, the soil enzyme synthesis function declines, microbial growth is restrained, soil organic matter decomposes at a slower pace, respiration is restrained, N fixation effects are reduced, and the turnover rate of C and N elements and the energy cycle are reduced; this results in lower soil organic matter and TN content. As a consequence, the soil water capacity, EC, soil nutrients, and other factors can change the soil enzyme activity, while coal mining increases the soil heterogeneity and causes changes to the soil enzyme activity. Changes in soil nutrients and uenzyme activity indicate that open pit mining leads to soil degradation, affecting the TOC, N, and P cycles of the soil, and thus affecting the total number and density of *T. mongolica*.

### 4.2. The Soil Bacterial Community Shift due to Open Pit Mining

Soil microorganisms are an important part of the rhizosphere soil ecosystem, and their quantity and distribution are influenced by many factors, which can reflect the subtle changes within the soil ecosystem. The secretion from the roots of plants have a serious effect on microorganisms of the rhizosphere. Soil microbial communities play an important role in ecosystems by regulating soil processes.

In the soil environments, bacteria species or strains are very abundant. The diversities are related to the vegetation, soil moisture, and depth. Open pit mining had severe effects on soil properties such as the SWC, soil EC, nutrient levels, and enzyme activities. Thus, the soil structure induced by open pit mining gradually decreases the bacterial richness and diversity. In the present study, the 7 dominant bacterial communities present in the soils belonged to the phyla Actinobacteria, Proteobacteria, Acidobacteria, Planctomycetes, Gemmatimonadetes, Bacteroidetes, and Chloroflexi in the same pattern observed from the above index. Among the bacteria from various topsoil sources, 92% belong to the 9 dominant phyla: Proteobacteria, Acidobacteria, Actinobacteria, Verrucomicrobia, Bacteroidetes, Chloroflexi, Planctomycetes, Gemmatimonadetes, and Firmicutes [31]. Li studied the effects of vegetation restoration among different soil bacterial communities, and the dominant phyla were Proteobacteria, Actinobacteria, and Acidobacteria [7]. Li [32] also investigated the diversity and composition of bacterial communities in response to reclamation of a soil subsidence area affected by mining activities, and found that the predominant phyla in present in the soils were Proteobacteria, Actinobacteria, Acidobacteria, and Planctomycetes. This indicates that the soil bacterial community in the *Tetraena mongolica* region is consistent with other types of soil at the phyla level.

In addition, the abundance of some core genera in the soil also changed substantially, such as the increase in *Sphingomonas*, *Gemmatimonas* and *Gaiella* and the decrease in *Nocardioides*. *Sphingomonas* sp. was a member of the hydrocarbon degrading family Sphingomonadaceae and relies on vitamins for its growth [33]. Vitamins are an essential micronutrient to promote biodegradation activity and accelerate the degradation of tributyl phosphate. Therefore, *Sphingomonas* was abundant in the contaminated area. *Sphingomonas* sp. Cra20 promotes the growth of *Arabidopsis thaliana* [34]. *Gemmatimonas* plays a key role in the fixation of organic carbon [35]. *Gemmatimonas* also participates in SOC dynamics because *Gemmatimonas* can metabolize fibers, thus promoting the degradation of cellulose [36]. Consequently, the *Gemmatimonas* content is higher in the topsoil. *Gaiella* and *Solirubrobacter* have also been reported to degrade polycyclic aromatic hydrocarbons [37]. *Nocardioides* species degrade aromatic amines by utilizing a mixture of aromatic amines as a source of carbon, nitrogen, energy, and sulfur [38]. *Nocardioides* sp. strain PD653 are capable of denitrating chloroaromatic compounds [39]. Acidobacterial subgroups Gp4 and *Gp6* are more abundant in soils with relatively high SOC [40]. It is reported that *Saccharibacteria_genera_incertae_sedis* mostly exists in desert areas [41]. Prediction of functional gene in rhizosphere soil showed that the largest differences between contaminated area and control area samples were the terms “Carbohydrate Metabolism” and “Peptidases”. The activity of all cysteine peptidases depends on a catalytic dyad of cysteine and histidine [42]. Rhizosphere soil increased the carbohydrate and amino acid metabolism, which were related to higher rates of soil C turnover [43]. Soil microbial richness and activity are closely related to the availability of soil nutrients. “Amino Acid Metabolism” were the energy and carbon sources of bacterial metabolism [44]. Thus, rhizosphere soil could promote more amino acid production and humic substance synthesis. During the metabolism, the microbes were accumulated in soil and probably concerned in further carbon and nitrogen cycling.

Open pit mining has no significant effect on the composition of the soil’s microbial community, but open pit mining reduces the diversity of soil bacteria, and the soil environment in the polluted area was poor, which changed the relative abundance of dominant bacteria. Thus, the dominant bacteria had strong anti-interference ability and open pit mining increased the abundance of bacteria for metabolism and degradation of organic pollutants. The dominant bacteria played an important role in nutrient cycling in poor soil. Microbial degradation is not only the main pattern of degrading mining area pollution but also one of powerful means to treat pollution in the environment.

### 4.3. Response of the Soil Bacterial Community to Changes in Soil Properties

Soil properties can affect the composition of bacterial communities, and those soil microbial communities play an important role in the ecosystem by regulating soil processes [9,45]. In this study, the correlation coefficient of CCA and Pearson’s correlation coefficients showed the relationship between the relative abundance of dominant flora and soil properties, indicating the response of the change of soil bacterial community to the change of soil biochemical characteristics. There was a significant correlation between the bacteria and the soil EC, SWC, TN, AP, and SOC, indicating that the soil bacterial community in Wuhai was also affected by the soil properties. Zeng [46] reported a significant correlation between soil bacterial communities and the levels of soil organic carbon, total nitrogen [24], and total phosphorus. Gao [47] observed that soil TP was positively correlated with Actinobacteria and negatively correlated with Firmicutes. Taylor [48] believed that soil enzyme activity was significantly correlated with soil microbial quantity and microbial diversity. Torres-Cortes found that the diversity of rhizosphere bacteria in central and southern Mexico changed with different seasons, and Acosta-Martínez identified positive correlations between Proteobacteria, Firmicutes, Chloroflexi, Verrucomicrobia, and Fibrobacteres and the activity of alkaline phosphatase and β-glucosidase or β-glucosaminidase [49]. Li studied the effects of vegetation restoration on the soil bacterial community, enzyme activity, and nutrients in the Loess Plateau and found that soil organic matter, available phosphorus, urease, and sucrase significantly increased, with the dominant phyla being Proteobacteria, Actinobacteria, and Acidobacteria [7]. Our result, among the main environmental factors, is that there is a significant relationship between every factor and soil bacterial community. Changes in dominant bacteria are related to soil properties, consistent with previous studies.

Thus, the changes of soil environmental factors and nutrients are the main factors controlling the changes of soil bacterial community structure. It may be because the area is located in the arid and semi-arid area, with less rainfall and large water evaporation. Microorganisms are involved in the decomposition of plant litter, and changes in the quality and quantity of litter lead to differences in soil TOC. However, open pit mining destroys the soil structure, which leads to the decrease of soil water holding capacity, the increase of electrical conductivity, and the accumulation of soil salt. The loss of water will be accompanied by the loss of nutrients. The accumulation of salt in the soil will hinder the nutrient cycle in the soil. Soil microbial communities play an important role in ecosystems by regulating soil processes. Therefore, the soil water and electrical conductivity are environmental factors that disturb the soil plants and microorganisms. This discovery indicates that open pit mining changes the physical and chemical properties of soil, decreases carbon and nitrogen cycling, and changes soil nutrients, which changes the structure of soil microbial communities in the *Tetraena mongolica* region.

## 5. Conclusions

Open pit mining leads to surface damage and deterioration of the ecological environment. Our study showed that open pit mining had severe effects on *Tetraena mongolica* soil properties such as the SWC, soil EC, nutrient levels, and enzyme activities. The results showed that the electrical conductivity (EC) increased; the TN, AP, AK, and SOC decreased, and the activity of four kinds of enzymes (NAG, sucrose, β-glucosidase, and alkaline phosphatase) in the soil decreased. In the present study, seven dominant phyla were found, in which the relative abundance of Actinobacteria, Proteobacteria, and Gemmatimonadetes increased while the abundance of Acidobacteria, Planctomycetes, Bacteroidetes, and Chloroflexi decreased. In addition, the abundance of some core genera in the soil also changed substantially, such as the increase in *Sphingomonas*, *Gemmatimonas,* and *Gaiella* and the decrease in *Nocardioides*. These results indicate that open pit mining caused the soil microbial community structure to shift, bacterial diversity and relative abundance to decrease, the carbon-nitrogen cycle to alter, the enzyme activity to decrease, the salinization of the soil to reach serious conditions, and the soil nutrient content to be reduced. This paper discusses the effects of open pit mining on *Tetraena mongolica* soil that were revealed; further studies are needed to investigate the harm brought by soil changes on *Tetraena mongolica*, and to reveal the potential interaction between plant community and soil microbial community.

## Figures and Tables

**Table 1:** Changes in plant community structure at sampling sites.

Sample Code	Vegetation Coverage (%)	Shannon- Index	Pielou’s Index	Simpson Index	Main Companion Species
P1	23.33	1.05	0.71	0.87	*Achnatherum splendens*
P2	28.49	1.23	0.72	0.89	*Reaumuria soongarica*
P3	21.29	1.18	0.69	0.90	*Achnatherum splendens*
H1	46.79	1.35	0.67	0.86	*Zygophyllum xanthoxylon*
H2	51.31	1.47	0.64	0.82	*Zygophyllum xanthoxylon*
CK	72.34	1.53	0.68	0.72	*Phragmites australis*

**Table 2:** Characteristics of soil bacterial richness and diversity indices in different soil samples.

Sample Code	Shannon	ACE	Chao1	Coverage	Simpson
P1-1	6.69	8139.96	7828.36	0.95	0.06
P1-3	5.93	7124.62	7228.36	0.95	0.09
P2-1	6.87	8496.79	7847.48	0.95	0.07
P2-3	5.84	7051.32	7025.39	0.95	0.10
P3-1	6.82	8308.25	7956.83	0.96	0.07
P3-3	5.75	7150.23	7081.62	0.97	0.09
H1-1	7.27	10,527.34	9096.68	0.96	0.05
H1-3	6.36	9488.99	8049.60	0.95	0.08
H2-1	7.15	11,035.31	8847.98	0.96	0.05
H2-3	6.56	9468.66	7766.70	0.95	0.07
CK-1	7.43	12,458.37	10,510.84	0.98	0.02
CK-3	6.71	10,703.70	9766.70	0.97	0.04

**Table 3:** Pearson correlations coefficient between the relative abundance of the dominant bacterial genera and environment factors.

	*Sphingomonas*	*Gp6*	*Gemmatimonas*	*Nocardioides*	*Gaiella*	*Aciditerrimonas*	*Saccharibacteria*
EC	0.135	0.463	0.643 *	−0.747 *	0.365	−0.201	−0.764 **
SWC	0.351	0.785 *	0.722 *	−0.678 *	0.146	−0.434	−0.763 *
SOC	0.576	0.414	0.347	−0.820 **	−0.106	−0.321	−0.797 **
TN	0.253	0.239	0.292	−0.839 **	0.055	0.023	−0.870 **
AP	0.602 *	−0.190	−0.191	−0.522	−0.317	−0.151	−0.485
AK	0.526	−0.112	−0.054	−0.581 *	−0.115	−0.177	−0.582
Sand	−0.139	−0.277	−0.508	0.576	−0.399	0.109	0.621 *

The correlations are significant at the 0.05 level. * indicates *p* < 0.05; ** indicates *p* < 0.01.
